# Optimization of yeast-based production of medicinal protoberberine alkaloids

**DOI:** 10.1186/s12934-015-0332-3

**Published:** 2015-09-16

**Authors:** Stephanie Galanie, Christina D. Smolke

**Affiliations:** Department of Chemistry, Stanford University, 443 Via Ortega, MC 4245, Stanford, CA 94305 USA; Department of Bioengineering, Stanford University, 443 Via Ortega, MC 4245, Stanford, CA 94305 USA

**Keywords:** Alkaloid, Plant natural product, Membrane protein, Cytochrome P450, *Saccharomyces cerevisiae*

## Abstract

**Background:**

Protoberberine alkaloids are bioactive molecules abundant in plant preparations for traditional medicines. Yeast engineered to express biosynthetic pathways for fermentative production of these compounds will further enable investigation of the medicinal properties of these molecules and development of alkaloid-based drugs with improved efficacy and safety. Here, we describe the optimization of a biosynthetic pathway in *Saccharomyces cerevisiae* for conversion of *rac*-norlaudanosoline to the protoberberine alkaloid (*S*)-canadine.

**Results:**

This yeast strain is engineered to express seven heterologous enzymes, resulting in protoberberine alkaloid production from a simple benzylisoquinoline alkaloid precursor. The seven enzymes include three membrane-bound enzymes: the flavin-dependent oxidase berberine bridge enzyme, the cytochrome P450 canadine synthase, and a cytochrome P450 reductase. A number of strategies were implemented to improve flux through the pathway, including enzyme variant screening, genetic copy number variation, and culture optimization, that led to an over 70-fold increase in canadine titer up to 1.8 mg/L. Increased canadine titers enable extension of the pathway to produce berberine, a major constituent of several traditional medicines, for the first time in a microbial host. We also demonstrate that this strain is viable at pilot scale.

**Conclusions:**

By applying metabolic engineering and synthetic biology strategies for increased conversion of simple benzylisoquinoline alkaloids to complex protoberberine alkaloids, this work will facilitate chemoenzymatic synthesis or de novo biosynthesis of these and other high-value compounds using a microbial cell factory.

**Electronic supplementary material:**

The online version of this article (doi:10.1186/s12934-015-0332-3) contains supplementary material, which is available to authorized users.

## Background

Alkaloids are nitrogen heterocycle-containing metabolites derived from amino acids. Alkaloids have wide-ranging chemical structures and bioactivities. Benzylisoquinoline alkaloids (BIAs) are a structural class of l-tyrosine derived plant alkaloids, including morphinan, protoberberine, and benzophenanthridine alkaloids. Protoberberine alkaloids occur in at least thirteen families of flowering plants, with the barberry, moon seed, poppy, and buttercup families comprising the most researched natural producers of these molecules [1]. Chinese, Indian, Islamic, and Native American traditional medicines all use plants in these families. Protoberberine alkaloids possess many reported bioactivities, including amelioration of multiple sclerosis in a mouse model [2], mitochondrially-mediated arrest of cell proliferation [3], and decreased blood sugar and cholesterol in diabetic patients [4].

Obtaining BIAs directly from their natural producers limits our ability to investigate the medicinal properties of many individual molecules and develop alkaloid-based drugs with improved efficacy, safety, and pharmacodynamics [5, 6]. First, only molecules that accumulate in crop plants are accessible. For example, morphine-producing cultivars of opium poppy accumulate morphine and codeine, but not upstream intermediates such as salutaridine. The concentrations of molecules that do accumulate vary with geography and climate, cultivation and harvest methods, plant material storage and processing, and the portions of the plant used. Second, purifying a molecule of interest from plant material requires intensive mechanical and chemical processing. Third, natural bioactive molecules often lack appropriate functional groups for derivatization to enable structure–activity studies.

Current alternatives to plant-based production of BIAs are plant cell culture and chemical or chemoenzymatic synthesis. For example, berberine is commercially produced via plant cell culture [7]. However, due to differences in enzyme expression, the *in planta* alkaloid profile differs from the plant cell culture alkaloid profile, and many alkaloids cannot be produced in cell culture. Furthermore, plant cell culture strains take longer to develop and have limited productivity relative to common industrial microbes because plant cells have longer generation times and are more difficult to genetically modify. Chemical or chemoenzymatic synthesis of BIAs enables production of bioactive molecules or intermediates of interest in a controlled, plant source-independent manner, effectively eliminating the challenges described above. However, the chemical synthesis of BIAs, including protoberberine alkaloids, is often challenging due to the presence of chiral centers, fused ring systems, and regiochemistry of ring substitutions and oxidations. This chemical complexity requires step-intensive routes, reducing yields [8]. In contrast, biocatalysis enables stereo- and regio-selectivity. For example, a chemoenzymatic route to (*S*)-scoulerine and (*R*)-reticuline included berberine bridge enzyme (BBE) to catalyze berberine bridge formation and kinetically resolve the (*R*)-enantiomer of reticuline [9].

Heterologous expression of multi-step biosynthetic pathways in a microbial host enables fermentative production or conversion of complex molecules in a single vessel, increasing yield by eliminating intermediate isolation steps. Engineered *Escherichia coli* cultures produced (*S*)-reticuline from dopamine [10] and later from glycerol [11] at titers up to 34 mg/L and productivity up to 0.57 mg/L/h [12]. The production of the protoberberine alkaloid (*S*)-scoulerine was also reported using a coculture of the reticuline-producing *E. coli* and *BBE*-expressing *S. cerevisiae* [10]. The downstream protoberberine (*S*)-canadine was produced from *rac*-norlaudanosoline (tetrahydropapaveroline) by an engineered yeast strain expressing seven heterologous genes encoding enzymes from *Thalictrum flavum*, *Papapver somniferum*, and *Arabidopsis thaliana* [13]. As a eukaryotic host organism, *S. cerevisiae* can support the functional expression of membrane-bound enzymes such as the flavoenzyme BBE and the cytochrome P450 oxidoreductase (P450) canadine synthase (CAS). Yeast strains can be efficiently engineered by in vivo assembly of episomal and chromosomally-integrated constructs, due to robust endogenous homologous recombination machinery. Furthermore, fermentation with engineered yeast is a scalable platform for production of complex plant alkaloids [5].

**Scheme 1:**
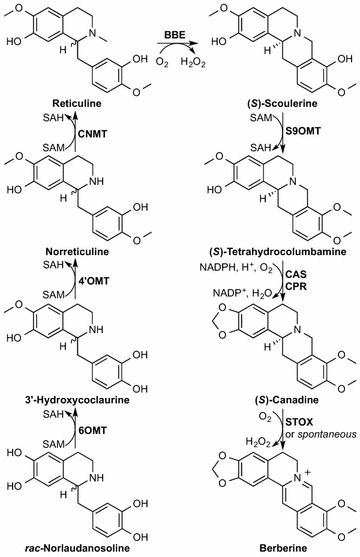
Heterologous berberine biosynthetic pathway from norlaudanosoline. Shown is the conversion of *rac*-norlaudanosoline to (*S*)-reticuline via the SAM-dependent methyltransferases norcoclaurine 6-*O*-methyltransferase (6OMT), 3′-hydroxy-*N*-methylcoclaurine 4′-*O*-methyltransferase (4′OMT), and coclaurine-*N*-methyltransferase (CNMT), and to the protoberberine alkaloids (*S*)-scoulerine via berberine bridge enzyme (BBE), (*S*)-tetrahydrocolumbamine via scoulerine 9-*O*-methyltransferase (S9OMT), (*S*)-canadine via canadine synthase (CAS) and its reductase partner cytochrome P450 reductase (CPR), and berberine via (*S*)-tetrahydroprotoberberine oxidase (STOX) or spontaneous oxidation

Here, we report the first heterologous production of berberine and the optimization of the engineered biosynthetic pathway from *rac*-norlaudanosoline to (*S*)-canadine in yeast (Scheme 1). The plant pathway includes three classes of membrane-associated enzymes: cytochrome P450 oxidorecuctase (P450), flavin-dependent oxidase, and cytochrome P450 reductase (CPR). We characterized P450 expression by fluorescence microscopy, optimized expression levels and stability via strain design and engineering, and used yeast codon-optimized enzymes from a variety of plant sources to increase protoberberine alkaloid titers. Media and culture condition optimization further increased titers. Overall, we increased the titer of (*S*)-canadine by greater than 70-fold relative to our previously reported strain, up to 1.8 mg/L, and were able to make berberine for the first time. A pilot scale 0.2-L bioreactor experiment resulted in titers of 0.7 mg/L canadine and 39 μg/L berberine. This (*S*)-canadine producing strain will enable heterologous biosynthesis of other protoberberine, berberine, and phthalide isoquinoline alkaloids for elucidating biosynthetic routes and, with further optimization, for industrial production.

## Results and discussion

### Cytochrome P450 expression engineering to improve (*S*)-canadine biosynthesis

Our laboratory previously engineered a *S. cerevisiae* W303α strain to biosynthesize (*S*)-canadine from the fed precursor (*R*, *S*)-norlaudanosoline [13]. This canadine-producing yeast strain (CSY410, Table 1) harbors expression cassettes for seven heterologous enzymes: *P. somniferum* norcoclaurine 6-*O*-methyltransferase (Ps6OMT, AAP45315), *P. somniferum* 3′-hydroxy-*N*-methylcoclaurine 4′-*O*-methyltransferase 2 (Ps4′OMT, AAP45314), *P. somniferum* coclaurine *N*-methyltransferase (PsCNMT, AAP45316), *P. somniferum* berberine bridge enzyme (PsBBE, AAC61839), *T. flavum* scoulerine 9-*O*-methyltransferase (TfS9OMT, AAU20770), *T. flavum* canadine synthase (TfCAS, AAU20771), and *A. thaliana* cytochrome P450 reductase 1 (CPR, AAK96879). The expression cassettes for the methyltransferases *Ps6OMT*, *PsCNMT*, and *Ps4′OMT* and the cytochrome P450 reductase *CPR* were chromosomally integrated, *TfS9OMT* and *TfCAS* were expressed from a high-copy plasmid (HCP), and *PsBBE* was expressed from a second HCP. All coding sequences were preceded by variants of the yeast *TEF1* promoter. To access berberine and other downstream products, such as noscapine, we optimized the (*S*)-canadine-producing strain.

**Table 1:** *S. cerevisiae* strains and plasmids used in this study

Plasmid	Description	Source
pCS6	pRS314, CEN/ARS vector, *TRP1* selectable marker	[50]
pCS8	pRS316, CEN/ARS vector, *URA3* selectable marker	[50]
pCS952	2 μ vector, P_*TEF1*_-*TfS9OMT*-T_*CYC1*_, *URA3* selectable marker	[13]
pCS953	2 μ vector, P_*TEF1*_-*TfS9OMT*-T_*CYC1*_, P_*TEF1*_-TfCAS-T_*CYC1*_, *URA3* selectable marker	[13]
pCS1018	2 μ vector, P_*TEF1*_-*PsBBE*-T_*CYC1*_, *TRP1* selectable marker	[13]
pCS3194	2 μ vector, P_*TDH3*_-repaired *TfCAS*-*EGFP*-T_*CYC1*_, *TRP1* selectable marker	This work
pCS3195	CEN/ARS vector, P_*TDH3*_-repaired *TfCAS*-*EGFP*-T_*CYC1*_, *TRP1* selectable marker	This work
pCS3196	CEN/ARS vector, P_*TDH3*_-*EGFP*-T_*CYC1*_, *TRP1* selectable marker	This work
pCS3197	2 μ vector, P_*TDH3*_-*EGFP*-T_*CYC1*_, *TRP1* selectable marker	This work
pCS3070	CEN/ARS vector, P_*TDH3*_-repaired *TfCAS*-T_*CYC1*_, *TRP1* selectable marker	This work
pCS3071	CEN/ARS vector, P_*TDH3*_-*TfCAS* from pCS952-T_*CYC1*_, *TRP1* selectable marker	This work
pCS3072	2 μ vector, P_*TDH3*_-*TfCAS* from pCS952-T_*CYC1*_, *TRP1* selectable marker	This work
pCS3409	CEN/ARS vector, P_*TEF1*_-repaired *TfCAS*-T_*CYC1*_, *TRP1* selectable marker	This work
pCS3100	YAC vector pYES1L, P_*PGK1*_-*PsBBE*-T_*PHO5*_, P_*TEF1*_-*TfS9OMT*-T_*CYC1*_, P_*TDH3*_-*TfCAS*-T_*ADH1*_, *TRP1* selectable marker	This work
pCS3198	YAC vector pYES1L, P_*PGK1*_-*PsBBE*-T_*PHO5*_, P_*TEF1*_-*yCjS9OMT*-T_*CYC1*_, P_*TDH3*_-*TfCAS*-T_*ADH1*_, *TRP1* selectable marker	This work
pCS3199	YAC vector pYES1L, P_*PGK1*_-*PsBBE*-T_*PHO5*_, P_*TEF1*_-*yPsS9OMT*-T_*CYC1*_, P_*TDH3*_-*TfCAS*-T_*ADH1*_, *TRP1* selectable marker	This work
pCS3200	YAC vector pYES1L, P_*PGK1*_-*PsBBE*-T_*PHO5*_, P_*TEF1*_-*yPsS9OMT*-T_*CYC1*_, P_*TDH3*_-*yPsCAS*-T_*ADH1*_, *TRP1* selectable marker	This work
pCS3201	YAC vector pYES1L, P_*PGK1*_-*PsBBE*-T_*PHO5*_, P_*TEF1*_-*yPsS9OMT*-T_*CYC1*_, P_*TDH3*_-*yAmCAS*-T_*ADH1*_, *TRP1* selectable marker	This work
pCS3202	YAC vector pYES1L, P_*PGK1*_-*PsBBE*-T_*PHO5*_, P_*TEF1*_-*yPsS9OMT*-T_*CYC1*_, P_*TDH3*_-*yCjCAS*-T_*ADH1*_, *TRP1* selectable marker	This work
pCS3203	YAC vector pYES1L, P_*PGK1*_-*PsBBE*-T_*PHO5*_, P_*TEF1*_-*yPsS9OMT*-T_*CYC1*_, P_*TDH3*_-*yCjCAS*-T_*ADH1*_, P_*TPI1*_-*yBwSTOX*-T_*STE2*_, *TRP1* selectable marker	This work
pCS3204	YAC vector pYES1L, P_*PGK1*_-*PsBBE*-T_*PHO5*_, P_*TEF1*_-*yPsS9OMT*-T_*CYC1*_, P_*TDH3*_-*yCjCAS*-T_*ADH1*_, P_*HXT7*_-*yBwSTOX*-T_*PGK1*_, *TRP1* selectable marker	This work

**Strain**	**Description**	**Source**
CSY3	W303 (*Mat*α*, ade2*–*1 ura3*–*1 his3*–*11, 15 trp1*–*1 leu2*–*3, 112 can 1*–*100*)	K. Weis
CSY288	CSY3 *his3*::P_*TEF1*_-*Ps6OMT*-T_*CYC1*_, *leu2*::P_*TEF1*_-*PsCNMT*-T_*CYC1*_, *ura3*::P_*TEF1*_-*Ps4′OMT*-T_*CYC1*_	[13]
CSY410	CSY288 *trp1*::P_*TEF1*_-*CPR*-T_*CYC1*_, pCS953, pCS1018	[13]
CSY835	CSY288 *trp1*::P_*TEF1*_-*CPR*-T_*CYC1*_	This work
CSY844	CSY288 *trp1*::P_*TEF1*_-*CPR*-T_*CYC1*_, *lys2*::P_*TEF1*_-*PsBBE*-T_*CYC1*_	[14]
CSY1005	CSY3, pCS3194	This work
CSY1006	CSY3, pCS3195	This work
CSY1007	CSY3 *fcy1*::P_*TDH3*_-*TfCAS*-*EGFP*-T_*CYC1*_, pCS6	This work
CSY1008	CSY3, pCS3196	This work
CSY1009	CSY3, pCS3197	This work
CSY1010	CSY3 *fcy1*::P_*TDH3*_-*EGFP*-T_*CYC1*_, pCS6	This work
CSY1011	CSY844, pCS6, pCS953	This work
CSY1012	CSY844, pCS952, pCS3072	This work
CSY1013	CSY844, pCS952, pCS3071	This work
CSY1083	CSY844, pCS952, pCS3409	This work
CSY1014	CSY844, pCS952, pCS3070	This work
CSY1016	CSY835, pCS8, pCS3100	This work
CSY1017	CSY844, pCS8, pCS3100	This work
CSY1018	CSY844, pCS8, pCS3198	This work
CSY1019	CSY844, pCS8, pCS3199	This work
CSY1020	CSY844, pCS952, pCS3199	This work
CSY1021	CSY844, pCS952, pCS3200	This work
CSY1022	CSY844, pCS952, pCS3201	This work
CSY1023	CSY844, pCS952, pCS3202	This work
CSY1024	CSY844 *XI*-*3*::P_*TEF1*_-yCjCAS-T_*CYC1*_, pCS952, pCS3202	XI-3 locus from [51] This work
CSY1025	CSY844, pCS952, pCS3203	This work
CSY1026	CSY844, pCS952, pCS3204	This work

To improve flux through the pathway, we first implemented two optimization strategies based on previous work in our laboratory [14]: increasing construct stability via integration and expressing the plant cytochrome P450 with an optimized regulatory profile. The first was implemented by integrating *PsBBE* into the chromosome instead of expressing from a HCP and the second by using the *TDH3* promoter (P_*TDH3*_) rather than the *TEF1* promoter (P_*TEF1*_) for P450 expression. Strains were assayed by analyzing the media supernatant by LC–MS/MS after 96 h culture with 2 mM norlaudanosoline. Reticuline, canadine, and berberine were identified by comparison of retention time and product ion spectra to authentic standards (Additional file 1: Fig. S1). The intermediates scoulerine and tetrahydrocolumbamine were identified by comparison of product ion spectra or MRM transitions to published LC–MS/MS analyses [15].

**Fig. 1:**
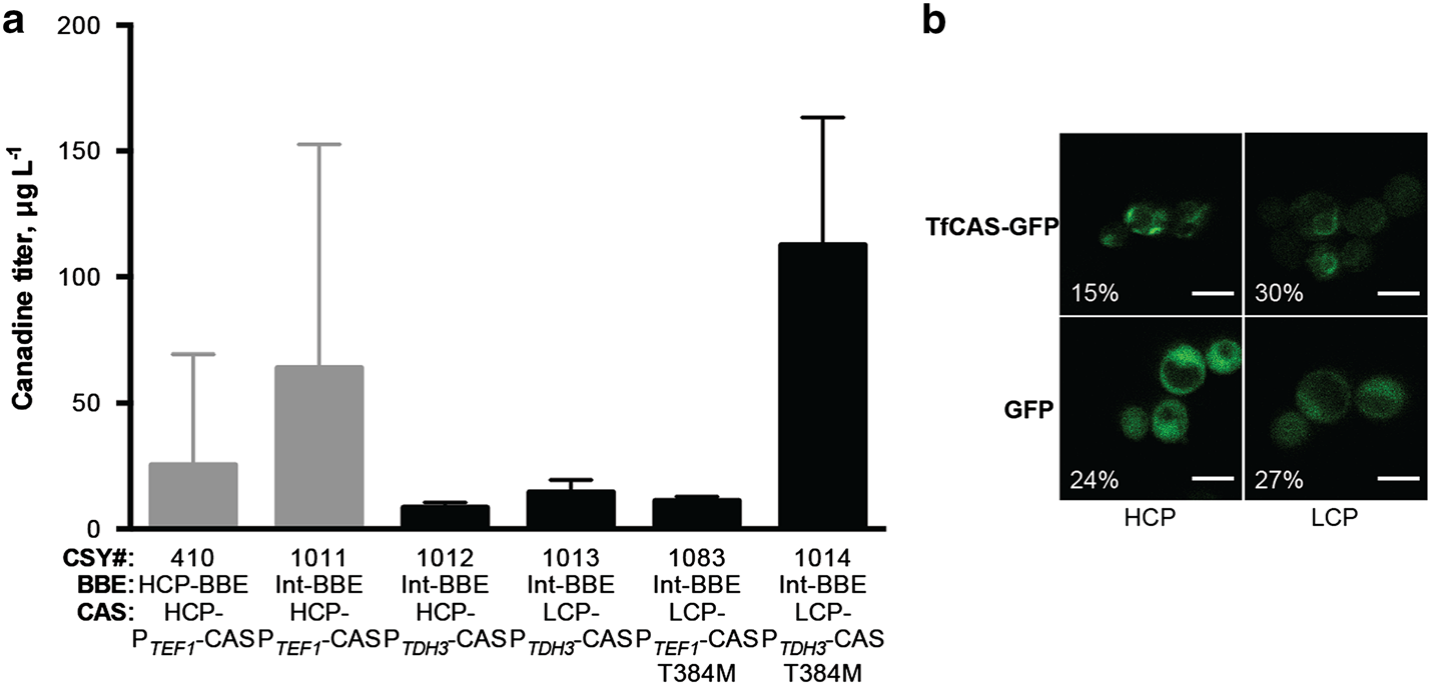
DNA copy number optimization and site directed mutagenesis of CAS for increased canadine titer. **a** (*S*)-canadine titers for *S. cerevisiae* expressing the protoberberine biosynthetic pathway with varied *TfCAS* constructs (Table 1). Int, chromosomally integrated; HCP, high copy plasmid; LCP, low copy plasmid. LC–MS/MS analysis was performed on media supernatant collected after 96 h of cultivation in YNB-DO (2 % dextrose) with 2 mM *rac*-norlaudanosoline.* Error bars* represent mean values ±1 SD of at least three biological replicates. **b** Confocal fluorescence microscopy images of TfCAS-GFP and GFP control. Percentages indicate the percent of cells that were fluorescent out of at least 300 cells counted from three separate clonal cultures. *Scale bars* 5 μm

Chromosomal integration of *PsBBE* slightly increased canadine production (CSY1011, Fig. 1a) relative to the original strain (CSY410), but with high variance. P_*TDH3*_-based expression of *TfCAS* on a HCP (CSY1012) decreased flux relative to P_*TEF1*_-based expression on a HCP (CSY1011). Canadine synthase is a microsomal heme-thiolate cytochrome P450 oxidoreductase that is N-terminally embedded in the endoplasmic reticulum (ER) membrane with the catalytic domain facing the cytoplasm [16]. The cytochrome P450 reductase, which transfers electrons from NADPH to the P450 in order to complete the heme catalytic cycle, is also N-terminally embedded in the ER. Therefore, properly localized expression is critical to heterologous microsomal P450 and P450 reductase activities, unless the enzymes are engineered to function without their localization domains. Live cell confocal fluorescence microscopy provided useful insights into the localization and qualitative expression level of a P450 in a similar multi-step heterologous pathway [14]. Thus, we imaged C-terminally EGFP-tagged TfCAS expressed from a HCP, low-copy plasmid (LCP), and chromosomal integration to determine TfCAS localization and expression levels (Fig. 1b). Neither the chromosomally integrated *GFP* control nor the integrated *TfCAS*-*GFP* construct was detectable above autofluorescence by fluorescence microscopy. Even though DNA copy number is equivalent for chromosomal integration and low-copy plasmid and genomic integration is more stable across the population, we have observed differences in fluorescence microscopy for GFP and other proteins in our laboratory, with chromosomal integration generally giving the same or lower fluorescence and activity. Additionally, we have seen differences in GFP fluorescence depending on the specific locus selected for integration. We believe the differences observed for GFP and TfCAS-GFP fluorescence between low-copy and integrated strains is due to lower expression from the integrated constructs resulting from the genomic context, chromosomal topology, and possible epigenetic silencing. With both LCP- and HCP-based expression, the GFP control fluorescence was detectable in 24–27 % of the imaged yeast cells. The TfCAS-GFP fusion was detected in a similar fraction of cells when expressed from a LCP, but only half as many as the control when expressed from a HCP. This suggests that high-copy expression of the P450 stresses the yeast host cell, which responds by reducing expression or eliminating the plasmid, despite growing the yeast under auxotrophic selective pressure.

Membrane-bound patterns of expression, which differ from the cytosolic expression of the GFP control, were observed for *TfCAS*-*GFP* expressed from both the HCP and LCP. However, small, highly saturated fluorescent regions were observed when *TfCAS*-*GFP* was expressed from a HCP in contrast to expression from a LCP. These saturated regions are likely indicative of protein aggregation or ER-associated degradation [17]. The imaging results suggest that lowering the expression level of *TfCAS* would increase the overall functional expression of the enzyme and therefore conversion efficiency from tetrahydrocolumbamine to canadine.

When *TfCAS* was expressed from P_*TDH3*_ on a LCP (CSY1013, Fig. 1a), canadine titer was increased 1.7-fold relative to HCP (CSY1012). These results are consistent with the hypothesis that the pattern of fluorescence observed from HCP expression through microscopy is detrimental to functional expression. Expression of *TfCAS* from a chromosomally integrated construct produced only trace levels of canadine, below the limit of quantitation. Similar to our observation for integrated *GFP* and *TfCAS*-*GFP*, we attribute this result to lower expression from the integrated construct relative to LCP. Additional improvement in canadine production was obtained by identifying an amino acid in TfCAS in the original yeast strain that differed from the deposited sequence and mutating this residue to correspond to the original sequence (CSY1014). In order to directly examine the effect of promoter choice in this context, we made a strain identical to CSY1014 with the *TEF1* promoter cloned in place of the *TDH3* for expression of the repaired *TfCAS*. We found that this new strain produced much less canadine than CSY1014, demonstrating that the *TDH3* promoter is better for *TfCAS* expression in this context. Taken together, these modifications resulted in a 4.4-fold increase in canadine titer relative to the original yeast strain (CSY410).

### Copy number and enzyme variant engineering to improve protoberberine alkaloid production

**Fig. 2:**
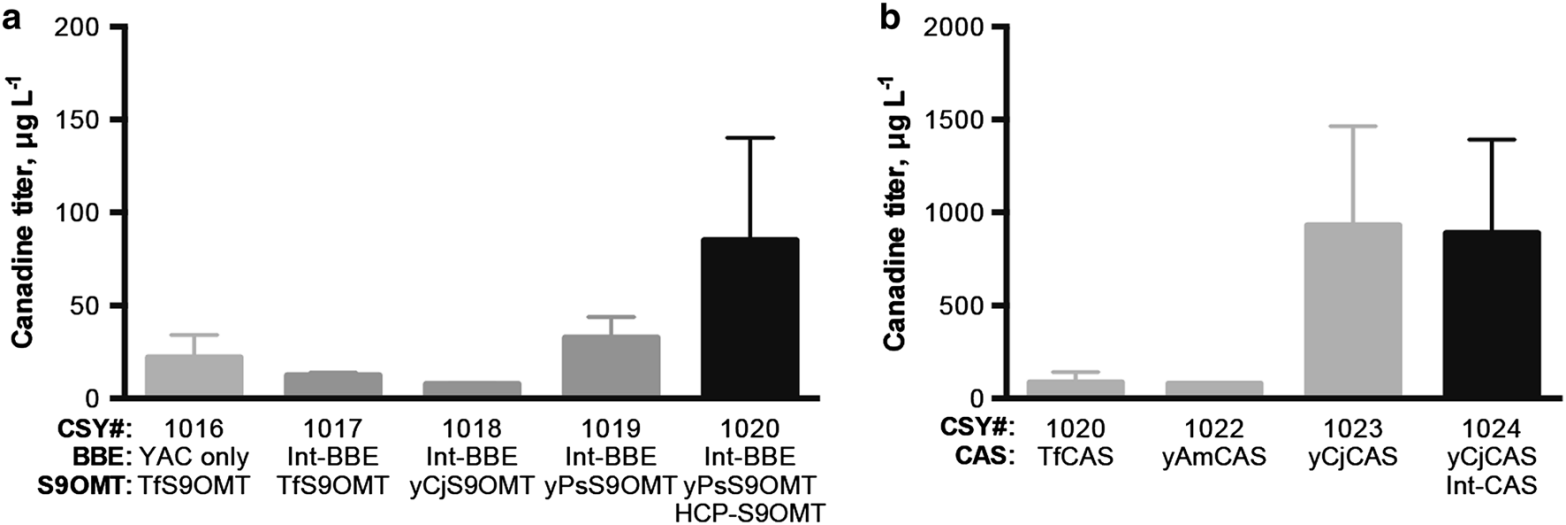
Enzyme variant and DNA copy number optimization of *S9OMT* and *CAS* for increased canadine titer. **a** *S9OMT*. **b** *CAS*. Strains listed in Table 1. LC–MS/MS analysis was performed on media supernatant collected after 96 h of cultivation in YNB-DO (2 % dextrose) with 2 mM *rac*-norlaudanosoline. *Error bars* represent mean values ±1 SD of at least three biological replicates

Approaches for tuning enzyme expression timing and levels, such as expression induction, promoter selection, and genetic copy number, can be used to balance flux through heterologous pathways. To reduce the number of auxotrophies needed for maintaining pathway enzymes, facilitate genetic copy number engineering, and simplify pathway assembly for rapidly testing various combinations of promoters and enzyme variants, we consolidated several pathway genes onto a yeast artificial chromosome (YAC). We anticipated that YACs would exhibit similar advantages as LCPs over HCPs for functional expression of cytochrome P450s and other membrane-bound enzymes, such as BBE. We rapidly assembled YACs in vivo in yeast from PCR-amplified expression cassettes and pYES1L backbone [18] using the strong glycolytic promoters P_*PGK1*_ and P_*TDH3*_ and the strong constitutive promoter P_*TEF1*_. The initial YAC, pCS3100, was assembled from P_*TEF1*_-*TfS9OMT*, P_*PGK1*_-*PsBBE*, and P_*TDH3*_-*TfCAS* cassettes in the strain with the *Ps6OMT*, *PsCNMT*, *Ps4′OMT*, and *CPR* expression cassettes chromosomally integrated (CSY1016). pCS3100 was also characterized in a similar strain background with a second copy of *PsBBE* chromosomally integrated (CSY1017). CSY1016 and 1017 produced less canadine than CSY1014 (Fig. 2a). Scoulerine accumulated in both of these strains (Additional file 1: Fig. S2), indicating that the TfS9OMT-catalyzed step was inefficient.

In CSY410, *TfS9OMT* was expressed from a HCP; thus we attributed the decreased canadine titers to the decreased expression of *TfS9OMT* on the YAC. To address the decreased S9OMT enzyme activity, we first examined whether other variants of the enzyme exhibit greater activity in the heterologous yeast host. We identified known orthologs by performing a protein BLAST search with TfS9OMT as the query (Additional file 2: Table S2). We replaced *TfS9OMT* in CSY1017 with yeast codon-optimized *S9OMT* genes from two other plants, *P. somniferum* (*yPsS9OMT*, AFB74611) and *Coptis japonica* (*yCjS9OMT*, Q39522). The *yPsS9OMT* strain CSY1019 produced 2.6-fold more canadine than the *TfS9OMT* strain CSY1017, whereas the *yCjS9OMT* strain CSY1018 produced less. As unconverted scoulerine was still observed in CSY1019 (Additional file 1: Fig. S2), an additional copy of *TfS9OMT* expressed from a HCP was added to the system to make strain CSY1020. CSY1020 exhibited less unconverted scoulerine, greater accumulation of the intermediate tetrahydrocolumbamine, yet little increase in canadine production (Additional file 1: Fig. S2).

To increase conversion from tetrahydrocolumbamine to canadine, we implemented a similar strategy to increase CAS activity, by examining enzyme variants from different plants for increased activity and additional gene copies for increased pathway flux (Additional file 2: Table S2). The yeast codon-optimized genes tested were from *C. japonica* (*yCjCAS*, Q948Y1), *Argemone mexicana* (*yAmCAS*, B1NF19), and *P. somniferum* (*yPsCAS*, ADB89214). The *yPsCAS* strain CSY1021 produced only trace amounts of canadine, indicating that this enzyme acts exclusively as a stylopine synthase and has little activity on tetrahydrocolumbamine [19]. The *yAmCAS* strain (CSY1022) produced about the same amount of canadine, and the *yCjCAS* (CSY1023) strain produced 11-fold more canadine than *TfCAS* strain CSY1020 (Fig. 2b). No further improvements in canadine titers were obtained by expressing a second copy of *yCjCAS* via chromosomal integration (CSY1024). While not all of the CAS enzyme variants that we assayed have been biochemically characterized, yCjCAS has the lowest *K*_*m*_ of the characterized variants, suggesting that it has the strictest substrate specificity [16]. Thus, the superior performance of the yCjCAS enzyme in our strains may be explained by increased substrate binding and specificity, and therefore increased pathway flux and reduced formation of side products.

### Spontaneous production of berberine

Berberine is used in traditional Chinese and Japanese medicine in its pure form and as part of *Coptis chinensis* or Huang Lian Su extract. Berberine is also found in *Hydrastis canadensis*, or goldenseal, and *Berberis aristata*, or Indian barberry, extracts used in Native American and Ayurvedic traditional medicines, respectively. Berberine is commercially produced by plant cell culture, but microbial biosynthesis can potentially exceed the productivity of plant cell culture due to decreased doubling times [7]. The enzyme that catalyzes the 4-electron oxidation of canadine to form berberine, (*S*)-tetrahydroprotoberberine oxidase (STOX), was recently cloned and characterized [20].

**Fig. 3:**
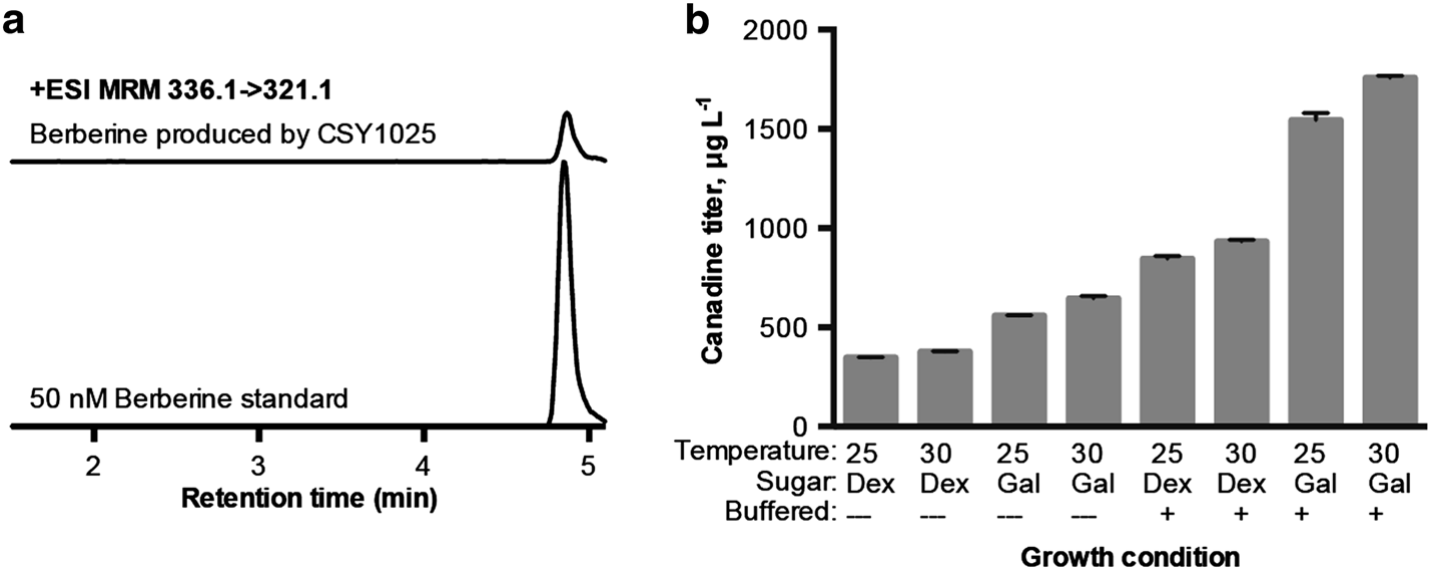
LC–MS/MS analysis and culture optimization of berberine-producing yeast strain. **a** LC–MS/MS chromatogram of berberine produced in the media supernatant of engineered strain CSY1025 after 96 h cultivation in YNB-DO (2 % dextrose) and of an authentic berberine standard. The peak heights are normalized to the height of the standard. **b** Effects of pH, sugar, and temperature on canadine titers. LC–MS/MS analysis was performed on media supernatant collected after 96 h of cultivation in YNB-DO (2 % dextrose) with 1 mM *rac*-norlaudanosoline in 12.5 mL shake flask cultures. *Error bars* represent range of two biological replicates

We assembled an expression cassette containing yeast codon-optimized *Berberis wilsonae* STOX (*yBwSTOX*) into pCS3203 with either the strong glycolytic promoter P_*TPI1*_ or the late-stage dextrose-repressed promoter P_*HXT7*_ to make strains CSY1025 and CSY1026, respectively. Following culture with norlaudanosoline, berberine was detected in the media from both strains, and its identity was confirmed by LC–MS/MS comparison to an authentic standard (Fig. 3a). Berberine was also detected at the same level in strain CSY1023 without *yBwSTOX*. We also observed gradual formation of berberine in canadine standards. We observe no significant increase in the strains with *STOX* relative to the strains without *STOX*, therefore all of the observed conversion could be attributed to non-enzymatic oxidation of canadine to berberine in aqueous solution. This non-enzymatic oxidation was also recently observed for the conversion of dihydrosanguinarine to sanguinarine [14, 21] and oxidation of 13,14-dihydrocoptisine to coptisine [22]. An earlier study showed that recombinant STOX expressed in Sf9 insect cells catalyzed conversion of canadine added directly to cell cultures to berberine [20], so we hypothesize that improving the functional heterologous expression of *STOX* in yeast would allow us to observe enzymatic oxidation, thereby increasing production of berberine. However, as the observed spontaneous formation of berberine suggests, canadine can also be readily converted to berberine chemically.

We implemented modifications to the host background that were directed to decreasing protein degradation rates by knocking out two endogenous yeast vacuolar protease genes, proteinase A (*PEP4*) and proteinase B (*PRB1*) [23]. Neither the single nor double knockouts improved canadine or berberine titers. We also examined the effect of lowering cytochrome P450 reductase (*CPR*) expression levels using the *TEF1* promoter mutant series [24] on canadine titers. We found that the wild type *TEF1* and *TDH3* promoters resulted in the highest canadine titers, with all of the lower expression mutant promoters decreasing titer (Additional file 1: Fig. S3). Therefore, strains CSY1023, 1025, and 1026 were able to convert the most *rac*-norlaudanosoline to (*S*)-canadine with a titer of 930 and <3 μg/L berberine titer after 96 h growth in culture tubes.

### Optimization of batch fermentation for protoberberine alkaloid production

To optimize batch fermentation conditions for greater protoberberine production and facilitate scale up, we explored the impact of pH, temperature, and sugar on canadine and berberine titers of strain CSY1025, which produced the most canadine. These experiments were performed in 12.5 mL cultures in shake flasks. The media pH was either allowed to acidify normally during cultivation or controlled by inclusion of 60 mM citrate buffer at pH 5.7. Temperature was held at 30 or 25 °C, and the carbon source was 2 % dextrose or 2 % galactose. The total canadine and berberine titers were quantified by LC–MS/MS by measuring the concentration in the media supernatant of cultures grown with 1 mM *rac*-norlaudanosoline. Buffering the media and using galactose as the sugar had the largest positive impact on titers, increasing the combined canadine and berberine titer 2.5-fold for buffered relative to unbuffered with dextrose and 1.9-fold for galactose relative to dextrose (Fig. 3b). Temperature did not significantly affect titers. With optimized culture conditions, the titers for canadine and berberine were 1.8 mg/L and 6.5 μg/L, respectively.

Maintaining higher pH conditions throughout the fermentation (5–5.7 for buffered media vs 3.5–5.7 for unbuffered media) likely improves titers because the plant enzymes have pH optima of 7.5–9 [15, 25–27]. Although yeast can grow at pH 7–7.5, the norlaudanosoline substrate is oxidatively unstable at pH above 6.5 due to the catechol moieties. Therefore, we anticipate that engineering yeast strains for de novo production, as was recently accomplished for the plant-derived alkaloid strictosidine [28], will allow for even greater product titers by eliminating the need to feed norlaudanosoline and allowing the process to be performed at neutral pH.

The positive impact of galactose on alkaloid titers of engineered yeast strains was previously noted [14]. Metabolizing galactose instead of dextrose changes gene expression in several yeast pathways, but it is not clear which of these effects are related to the improved titers [29]. Galactose can also be converted into l-ascorbic acid by yeast [30], and the antioxidant ascorbic acid was previously reported to stabilize norlaudanosoline in *E. coli* cultures [31]. However, including 10 mM ascorbic acid in culture media did not impact product titers (data not shown).

### Pilot scale batch and fed-batch cultivation of protoberberine-producing strain

**Fig. 4:**
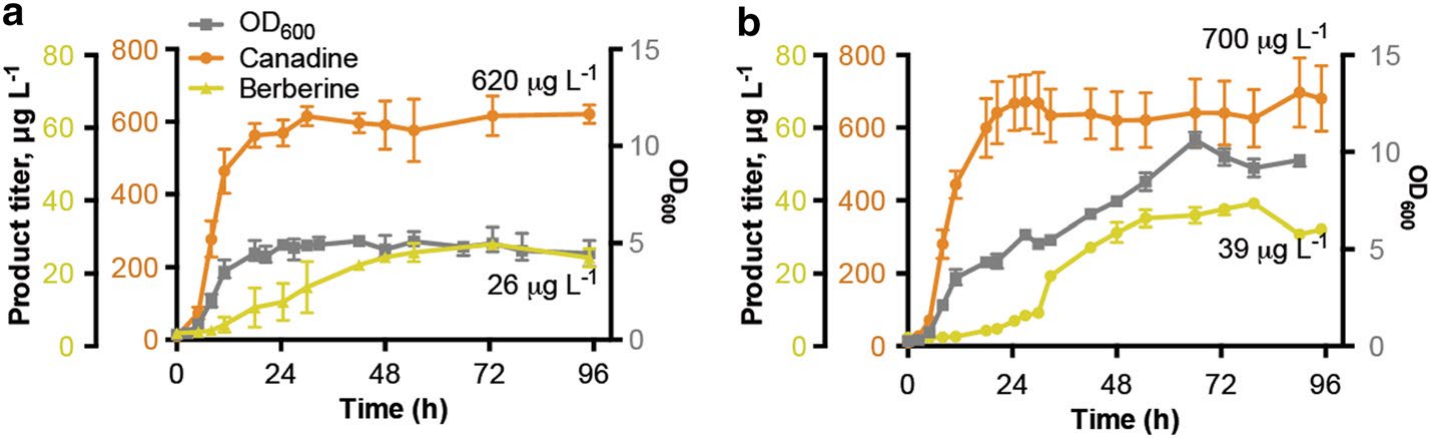
Pilot scale fermentation of engineered *S. cerevisiae* strain CSY1025. **a** Pilot scale batch and **b** fed-batch 0.2-L fermentations in bioreactor with YNB-DO (2 % dextrose) with 1 mM *rac*-norlaudanosoline. Canadine and berberine titers were determined by LC–MS/MS from sample supernatant at indicated time points. *Error bars* represent the range of two independent fermentations

To further evaluate the engineered strain CSY1025 for protoberberine production from *rac*-norlaudanosoline, 0.2-L pilot scale batch fermentations were performed in a bioreactor. Based on results from the shake flask experiments, citrate buffer was included in the media and 30 °C growth temperature was maintained. Despite the improvement observed with galactose in shake flasks, we used dextrose in the bioreactor runs because of its greater ability to support rapid biomass accumulation. Final titers of 621 μg/L canadine and 27 μg/L berberine were obtained after 72 h of batch cultivation with a final OD of only 5.2 (Fig. 4a). This canadine titer was 67 % of what was observed in culture tubes and shake flasks, and the berberine titer was nearly tenfold higher than what was observed in tubes and flasks. Canadine production was highly growth associated, so we investigated whether increasing strain growth would also increase canadine production by performing a fed-batch fermentation.

Because we observed low ODs in the batch fermentation, we hypothesized that biomass was limiting to substrate conversion. For fed-batch conditions, 10× of the same media without buffer was fed at a rate of 20 mL/day starting at 24 h. Fed-batch conditions increased the strain OD up to 10.6 with a 13 % increase in canadine titer and a 44 % increase in berberine titer (Fig. 4b). Given the variation between fermentations, there is not a significant difference between the canadine titers obtained in batch relative to fed-batch. When dilution by the feed volume is taken into account, this corresponds to a 41 % increase in the overall amount of canadine and an 81 % increase in the amount of berberine. Since increasing the ODs by fed-batch fermentation did not significantly improve titers, we concluded that the specific productivity of the strains, rather than or jointly with the biomass concentration, requires further optimization. The lack of correlation between conditions that yield high biomass and conditions that yield high product titers has been observed previously [32]. Further increases in canadine titer may be obtained by engineering the yCjCAS enzyme to relieve any product inhibition, making further changes to yeast metabolism to obtain redox balance, and engineering the heterologous pathway and native metabolism to reduce any diversion of canadine to other side products. The spontaneous formation of berberine from canadine was low (<7 %), and is increased in the bioreactor relative to shake flasks, likely due to increased oxygen transfer rate for the bioreactor relative to flasks. A larger reactor would have a reduced oxygen transfer rate, and aeration can be further tuned by modulating stir speed and purging rate. Therefore, we do not anticipate spontaneous formation of berberine to be a major limitation to producing canadine derivatives. Improved functional expression of *yBwSTOX* would increase flux to berberine, and connecting this pathway to upstream metabolism for de novo production would allow us to fully optimize carbon use and redox balance to maximize flux through the pathway.

The current optimized yeast strain with 70-fold higher titer of (*S*)-canadine would result in ~70-fold decrease in process cost relative to the previously reported yeast strain [13]. For plant cell culture, the estimated break even point for a product selling cost, inflation adjusted, is ~$2800/kg [33]. Berberine currently sells for ~$6000/kg and (*S*)-canadine is only available as a specialty chemical, selling for ~$4800/g. The industrial plant cell culture process is based on a fed-batch culture of *Coptis japonica* cells, which can reach a berberine titer of 3.5 g/L after 14 days of culture [34]. (*S*)-canadine, of value for stereospecific synthesis of phthalideisoquinoline alkaloids, is not accessible by plant culture because of high conversion to berberine. At these selling price points, a microbial fermentation titer of 1–2 g/L is needed for production of berberine, and even lower for canadine [35]. The yeast process reported here produces 1.8 mg/L canadine after 4 days of culture. Combining the engineered strain reported here with a strain reported to produce (*S*)-reticuline from sugar [36, 37] and conducting further research for the necessary strain, pathway, and fermentation improvements to obtain a canadine titer of 60–200 mg/L, similar to the industrial processes for paclitaxel and vitamin B_12_ [38, 39], could result in an industrially relevant process. Additionally, establishing the plant cell culture inoculum takes up to a month, while a yeast inoculum requires only 24 h [40]. In plant cell culture, alkaloids accumulate in vacuoles, so downstream processing requires cell disruption, solid–liquid separation, and product recovery. In yeast culture, the alkaloids accumulate in the media rather than in the cells, so cell disruption is not required, reducing processing by one unit operation. Overall, with further development yeast fermentation could achieve cost savings by decreasing fermentation time and simplifying downstream processing.

## Conclusions

In this work, we optimized the reconstituted seven-enzyme pathway, including three types of membrane-bound enzymes, to convert the commercially available and synthetically accessible substrate *rac*-norlaudanosoline to the protoberberine alkaloid (*S*)-canadine and the medicinal compound berberine. We optimized this pathway by examining the effects of DNA copy number, enzymes from different plant sources, and culture conditions. Consistent with previous reports, we observed greater cytochrome P450 activity when expressed from 1 to 2 DNA copies with a glycolytic promoter [14]. Ultimately, the best yields were obtained by using enzymes from a variety of plant sources and combining YAC-, chromosome-, and high-copy plasmid-based expression. The strain and culture engineering resulted in an over 70-fold increase in the canadine titer relative to the original strain, and we demonstrated that similar titers could be achieved in a pilot scale bioreactor. (*S*)-canadine is useful as a precursor for a number of chiral alkaloids, including the phthalideisoquinoline alkaloid noscapine, a potential anticancer drug [41]. This work also describes the first heterologous production of berberine, which was produced at more than tenfold greater titer in the bioreactor than in shake flasks or culture tubes. Our combined metabolic engineering and synthetic biology approach for in vivo microbial reconstitution of alkaloid biosynthesis is a useful tool for biochemical discovery and fermentative production of complex, high-value molecules.

## Methods

### Chemicals, media, and cultivation

Difco yeast nitrogen base without amino acids and ammonium sulfate (YNB), Bacto peptone, Bacto yeast extract, Luria Broth (LB), LB agar, dextrose, and galactose were obtained from Becton, Dickinson and Company (BD). Kanamycin monosulfate, geneticin sulfate (G418), ampicillin, spectinomycin, amino acids, uracil, adenine hemisulfate, tris(hydroxymethyl)aminomethane hydrochloride, polysorbate 20 (Tween-20), and citric acid were obtained from EMD chemicals. 5-fluoroorotic acid (5-FOA) was obtained from Zymo Research. Formic acid, acetonitrile, NuPAGE 3-(*N*-morpholino)propanesulfonic acid (MOPS) sodium dodecyl sulfate (SDS) running buffer, and NuPAGE transfer buffer were obtained from Thermo Fisher Scientific. 1-(3,4-dihydroxybenzyl)-1,2,3,4-tetrahydroisoquinoline-6,7-diol hydrobromide (tetrahydropapaveroline or norlaudanosoline, NL) and berberine hydrochloride were obtained from Santa Cruz Biotechnology (SCBT). (*S*)-reticuline perchlorate was obtained from Specs and d,l-canadine was obtained from Apin Chemicals.

*Escherichia coli* strains were selected on LB agar plates with 50 mg/L kanamycin, 50 mg/L ampicillin, or 100 mg/L spectinomycin and grown in LB liquid media with the appropriate antibiotic. Yeast 10× drop out (DO) supplement was prepared as 0.2 % adenine hemisulfate, 0.2 % uracil, 1 % l-tryptophan, 1 % l-histidine hydrochloride, 1 % l-arginine, 1 % l-methionine, 0.2 % l-tyrosine, 1 % l-leucine, 1 % l-isoleucine, 1 % l-lysine hydrochloride, 1 % l-phenylalanine, 1 % l-glutamic acid, 1 % l-aspartic acid, 3 % l-valine, 4 % l-threonine, and 8 % l-serine with the desired selection component omitted. *S. cerevisiae* strains were selected on YNB-DO (0.17 % yeast nitrogen base, 0.5 % ammonium sulfate, 2 % dextrose, and 1× DO) agar or on YPAD (1 % yeast extract, 2 % peptone, 80 mg/L adenine hemisulfate, and 2 % dextrose) agar with 200 mg/L G418. Yeast were grown in selective YNB-DO media or in YPAD media.

### Strains and plasmids

*Escherichia coli* strain TOP10 (Life Technologies) was used for cloning and amplification of plasmids. Plasmids were recovered using Econospin columns (Epoch Life Sciences) according to manufacturer’s instructions. *S. cerevisiae* strain W303α was used as the base strain for all engineered strains (Table 1). Oligonucleotide primer sequences are provided in Additional file 2: Table S1. Oligonucleotides were synthesized by Integrated DNA Technologies (IDT) and the Stanford Protein and Nucleic Acid Facility. Heterologous gene sequences were cloned from previously published plasmids or downloaded from Genbank and codon optimized and synthesized by Life Technologies or IDT (Additional file 2: Table S2). PCRs were performed with PfuUltraII Fusion HS DNA polymerase (Agilent Technologies) for <2 kb fragments, Platinum Taq PCR SuperMix (Life Technologies) for 2–3 kb fragments and for site-directed mutagenesis, or Expand High Fidelity PCR system for >3 kb fragments (Roche Diagnostics) according to manufacturer’s instructions. PCR products were purified by agarose gel extraction with Zymoclean gel DNA recovery kit (Zymo Research) according to manufacturer’s instructions. Restriction enzymes, T4 DNA ligase, and deoxynucleotides were purchased from New England Biolabs.

Strains and plasmids used or constructed in this work are described in Table 1. Plasmids pCS3070–3072 and 3194–3197 were constructed by amplifying the insert with either a CACC 5′ overhang or 5′ and 3′ BP sequences and using the pENTR/D-TOPO cloning kit or Gateway BP clonase II and pDONR221 (Life Technologies), respectively, to create a Gateway entry vector. The insert was then cloned into a pAG destination vector obtained from Addgene [42] using Gateway LR clonase II (Life Technologies). Constructs were verified by sequencing through the inserted region (Elim Biopharmaceuticals). Plasmids were introduced into yeast by the lithium acetate/salmon sperm carrier DNA/polyethylene glycol transformation method [43].

Holding vectors were constructed by amplifying the backbone, including the promoter and terminator, and assembling with the insert via Gibson cloning [44]. Expression cassettes were amplified using primers with 15 bp overhangs for gap repair in yeast. 100 ng each of the PCR products and linearized pYES1L (Life Technologies) were introduced by electroporation into the desired yeast background strain to make constructs pCS3100, 3198, 3199, and 3200–3204 [18, 45]. Plasmids were isolated from yeast and amplified in *E. coli* as previously described.

Yeast chromosomal modifications were made using either disintegrators [46] or the microhomology gene disruption method [47]. For strains CSY1007 and CSY1010 the expression cassette was cloned from plasmid pCS3070 and pCS3196, respectively, into the KpnI–SphI restriction sites of plasmid pIS373 [46]. For strains CSY1024, the yCjCAS coding sequence was inserted between the *TEF1* promoter and *CYC1* terminator by LR recombination into plasmid pCS2641, which is pUG6 with the promoter and terminator cassette cloned in along with the Gateway recombination sites. The disintegrators or gene disruption cassettes were introduced into yeast by lithium acetate transformation [43]. All selection markers were rescued as previously described [46, 47]. For the disintegrators, this was done by diluting an overnight culture to OD600 ~0.4 and plating 250 μL of the cell suspension on YNB-DO agar with 1 mg/mL 5-FOA. For the microhomology integrations and knockouts, selection markers were rescued by loxP recombination. All modifications were verified by yeast colony PCR as previously described, with the exclusion of gelatin from buffers [48].

### Confocal microscopy

Single colonies of freshly transformed yeast strains were streaked onto YNB-DO agar, grown for 24 h, and then inoculated into 3 mL of the appropriate YNB-DO media and grown in 16 × 150 mm glass culture tubes for 14 h at 30 °C in a shaking incubator at 260 rpm. Overnight cultures were back-diluted 1:2 in YNB-DO media and grown for an additional 2 h to OD600 ~2. 1 mL of the cell culture was pelleted and resuspended in 100 μL YNB-DO. 1 μL of the resuspended cells was mounted on 2 % low melting point agarose YNB-DO pads with a no. 1.5 coverslip sealed with nail polish. Live yeast were imaged with a Leica SP5 confocal upright microscope at the Stanford Cell Sciences Imaging Facility equipped with a 63×, NA 1.30 plan apochromat glycerine immersion objective. For fluorescence detection, the Argon laser 488 nm excitation was used with descanned Hybrid-GaAsP detection at 500–600 nm. Imaging was done with either no zoom or 4.5× zoom and 80–120 % gain to avoid saturation.

### Yeast culture assays for protoberberine alkaloid production

Single colonies of freshly transformed yeast strains were streaked onto YNB-DO agar, grown for 24 h, and then inoculated into 3 mL of the appropriate YNB-DO media and grown in 16 × 150 mm glass culture tubes for 17 h at 30 °C in a shaking incubator at 260 rpm. Overnight cultures were back-diluted 1:20 into assay media composed of YNB-DO media with 2 mM norlaudanosoline HBr (NL) to a total volume of 500 μL in culture tubes. Cultures were grown for 96 h and then pelleted by 5 min centrifugation. The supernatant was analyzed by high performance liquid chromatography–tandem mass spectrometry (LC–MS/MS). The optimization assays were performed with 1 mM norlaudanosoline HBr in 125-mL flasks in a total volume of 12.5 mL assay media with 2 % galactose or 2 % dextrose used as the sugar, either no buffer or 60 mM citrate buffer pH 5.7 was added, and cultures were grown at 25 or 30 °C. To determine the canadine and berberine titers of the cultures, 1 mL of culture was pelleted and the supernatant was analyzed by LC–MS/MS as described below.

### Analysis of protoberberine alkaloid production by high-performance liquid chromatography–tandem mass spectrometry (LC–MS/MS)

Yeast media supernatants and yeast cell extracts were analyzed by LC–MS/MS using an Agilent 1260 infinity binary pump HPLC and Agilent 6420 triple quadrupole mass spectrometer with an electrospray ionization source. Chromatography was performed with a Zorbax Eclipse Plus C18 column (2.1 × 50 mm, 1.8 μm, Agilent Technologies). Mobile phase A was water with 0.1 % formic acid (FA), phase B was acetonitrile with 0.1 % FA, and the flow rate was 0.4 mL/min at 40 °C. 5 μL samples were injected and separated with the following method: 10 % B for 0.1 min, 10–40 % B for 4.9 min, 40–98 % B for 1 min, held at 98 % B for 1 min, returned to 10 % B and re-equilibrate for 3 min. The LC eluent was directed to the MS for 1–5 min with ESI source gas temperature 350 °C, gas flow of 11 L/min, nebulizer pressure 40 PSI, capillary voltage of 4000 V, and Delta EMV (+) of 200. Compound identity was confirmed by comparing the retention time and product ion spectrum to an authentic standard when available or to published mass fragmentation spectra [15, 49] using MassHunter Qualitative Analysis v. B.06 (Agilent Technologies). For quantification, the MS was used in MRM mode to monitor the transitions in Table 2. These transitions were determined using the MassHunter Optimizer software with standards when available or with samples with high abundance and confirmed with literature. The data was analyzed using MassHunter Quantitative Analysis v. B.07 for QQQ (Agilent Technologies) to identify and integrate peaks with the qualifying and quantifying mass transitions and retention times (Table 2). Where available, quantifier MRM peak areas were compared to a calibration curve of external standard peak areas to determine concentration.

**Table 2:** MRM transitions used to quantify alkaloids in LC–MS/MS analysis

Compound	Quantifier MRM transition			Qualifier MRM transition		
	Precursor → product ion	Fragmentor	Collision energy	Precursor → product ion	Fragmentor	Collision energy
Reticuline	330 → 192	120	19	330 → 137	120	31
Scoulerine	328 → 151	135	30	328 → 178	135	29
Tetrahydrocolumbamine	342 → 178	135	29	342 → 163	135	29
Canadine	340 → 176	135	29	340 → 149	135	25
Berberine	336 → 320	135	29	336 → 292	135	25

### Batch and fed-batch fermentations of engineered yeast strains

Batch and fed-batch fermentations were conducted in a Biostat Q-plus bioreactor with 0.5-L univessels (Sartorius AG). Initial media was 0.2 L YNB-DO with 1 mM norlaudanosoline HBr and 60 mM citrate buffer, pH 5.7. Single colonies of freshly transformed yeast strains were streaked onto YNB-DO agar, grown for 24 h, and then inoculated into 3 mL of the appropriate YNB-DO media and grown in 16 × 150 mm glass culture tubes for 24 h at 30 °C in a shaking incubator at 260 rpm. Cultures were back-diluted 1:10 in 25 mL YNB-DO in 250-mL shake flasks and grown to OD 3.5–7, and then used to inoculate the bioreactor vessels to OD 0.3. Dissolved oxygen was calibrated at 100 % during 100 rpm stirring with 0.1 lpm air flow. Process parameters were 30 °C and 25 % dissolved oxygen controlled by stirring at 100–400 rpm. Fermentation was begun with no air flow, and air flow was increased to 0.1 lpm when stirring reached 400 rpm, 12–24 h. When stir rate dropped to 100 rpm and DO reached 80 %, air flow was turned off. Samples of ~1 mL were taken to measure OD_600_ and alkaloid concentrations. OD_600_ of diluted culture samples was measured in cuvettes by Nanodrop spectrophotometer. For fed-batch, feed was 10× YNB-DO media, and feeding was begun at 24 h at a rate of 20 mL/day, and continued for the remainder of the fermentation.

## Additional files

**Additional file 1.**

**Additional file 2.**
