# Supplementary material for: Optimization of yeast-based production of medicinal protoberberine alkaloids
Source: Microb Cell Fact. 2015 Sep 16;14:144. doi: 10.1186/s12934-015-0332-3 (PMC4574094; doi:10.1186/s12934-015-0332-3)
Supplement: Supplementary file 1 — Additional file 1. [file 12934_2015_332_MOESM1_ESM.pdf]

Supplementary Figures

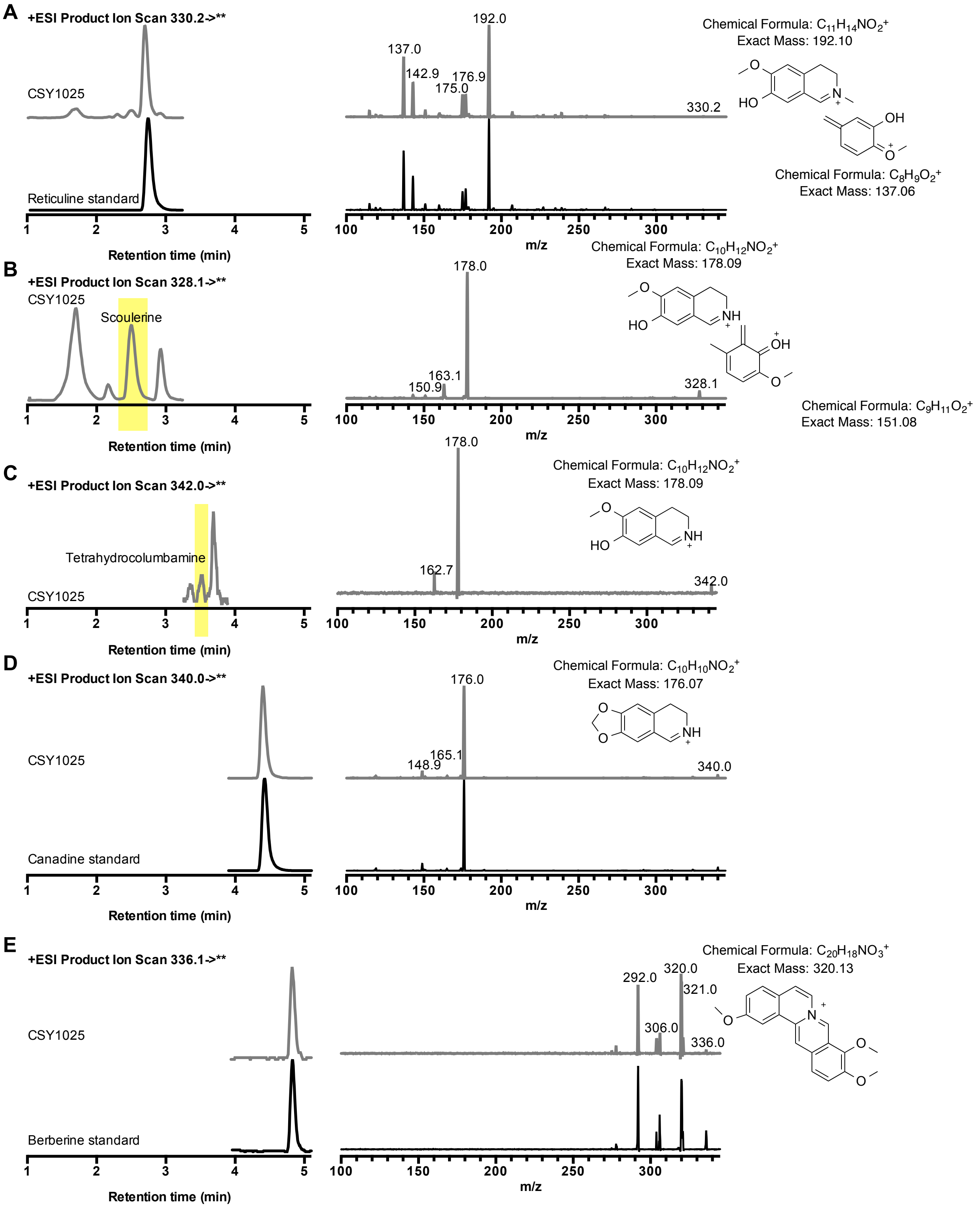

Fig. S1. LC-MS/MS chromatograms and mass fragmentation patterns of authentic standards and product molecules. (A) Top, reticuline produced by engineered yeast; bottom, authentic (*S*)-reticuline standard. (B) Scoulerine produced by engineered yeast. (C) Tetrahydrocolumbamine produced by engineered yeast. (D) Top, canadine produced by engineered yeast; bottom, authentic (*D,L*)-canadine standard. (E) Top, berberine produced by engineered yeast; bottom, authentic berberine standard. Samples were taken from pilot scale batch fermentation of strain CSY1025 at 24 (A-D) and 33 h (E), and the supernatant was analyzed by LC-MS/MS. Product ion spectra were obtained with a collision energy of 30 V. Chromatograms and mass spectra are normalized such that the highest peak is 100%.

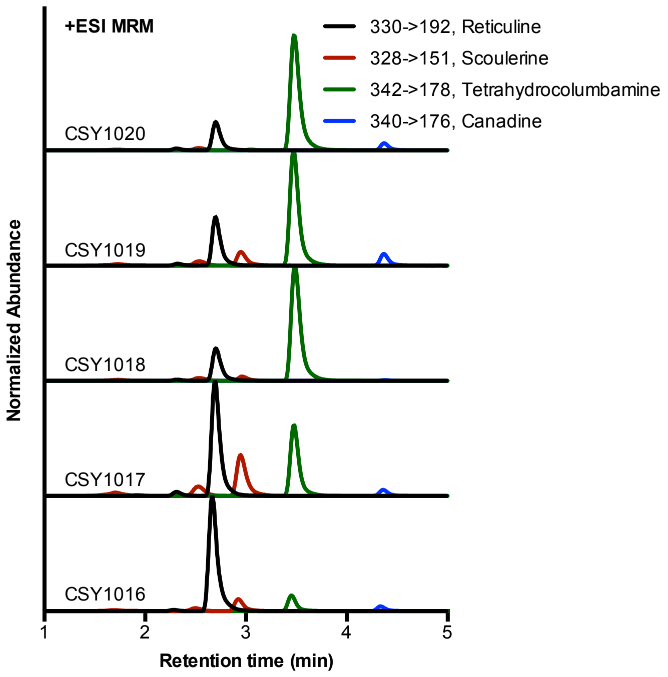

Fig. S2. LC-MS/MS chromatograms of strains SGY12-16 showing accumulation of reticuline, scoulerine, tetrahydrocolumbamine, and canadine. Strains are described in Table 1. LC-MS/MS analysis was performed on media supernatant collected after 96 h of cultivation in YNB-DO (2% dextrose) with 2 mM *rac*-norlaudanosoline. Each chromatogram is from a single representative sample of at least three and normalized such that the highest peak is 100%.

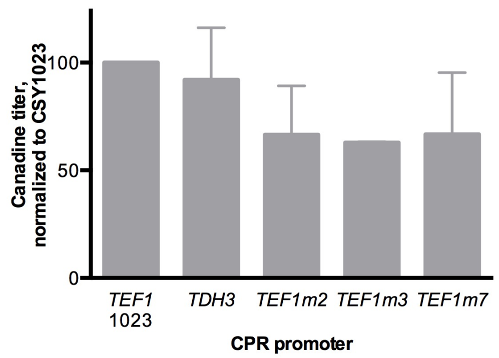

Fig. S3. Promoter optimization of integrated *CPR* construct in CSY1023 for increased canadine titer. LC-MS/MS analysis was performed on media supernatant collected after 96 h of cultivation in YNB-DO (2% dextrose) with 2 mM *rac*-norlaudanosoline. Error bars represent range of 2 biological replicates. Mutant *TEF1* promoters are from Nevoigt et al., 2006.
